# Supplementary material for: Electronic Nose and Head Space GC–IMS Provide Insights into the Dynamic Changes and Regularity of Volatile Compounds in Zangju (Citrus reticulata cv. Manau Gan) Peel at Different Maturation Stages
Source: Molecules. 2023 Jul 11;28(14):5326. doi: 10.3390/molecules28145326 (PMC10384022; doi:10.3390/molecules28145326)
Supplement: Supplementary file 1 [file molecules-28-05326-s001.zip › molecules-2436845-supplementary.pdf]

	Compounds	ZGP-1	ZGP-2	ZGP-3	ZIP-1	ZIP-2	ZIP-3	ZJP-1	ZJP-2	ZJP-3	ZKP-1	ZKP-2	ZKP-3	ZLP-1	ZLP-2	ZLP-3
**Alcohols**																
1	1-Hexanol	406.2113	395.72433	440.48123	701.60803	1058.2347	821.4178	757.9723	687.11224	699.7671	256.62918	455.16742	435.74875	912.42017	891.2336	792.80084
2	Benzyl alcohol	472.30716	489.74203	483.57492	467.84448	474.694	471.374	476.75076	478.1886	489.43417	372.38577	417.83777	433.49203	495.99805	477.69662	402.89764
3	1-Penten-3-ol	602.42615	604.4099	578.3639	506.6818	435.47897	488.9549	293.20978	246.56119	128.0813	354.78583	565.9662	507.04047	644.8057	799.5234	774.65814
4	2-Methyl-1-pentanol	19.215528	23.497284	19.301228	22.621254	22.564121	19.694805	20.177258	21.894403	19.536104	170.099	176.47562	190.74919	224.09546	240.39725	195.21822
5	2-Methyl-1-butanol M	126.70378	144.14503	148.6458	87.31099	86.02869	95.46506	102.10511	94.64616	101.10212	478.1632	505.34872	510.5922	568.80695	586.0038	556.8885
6	2-Methyl-1-butanol D	55.39303	70.61564	60.284195	29.26448	39.55466	28.667765	35.4443	38.672283	32.209972	268.1731	315.05658	339.56003	431.0385	438.799	377.51498
7	3-Methyl-1-butanol	61.141182	65.97522	70.26015	46.63273	42.49698	44.10621	44.096684	43.91894	40.929012	113.6395	120.0542	118.95282	116.02637	155.11761	158.03455
8	1-Butanol	90.129524	86.93963	98.715256	105.43148	147.21431	109.427574	96.026855	87.11738	110.05286	316.25635	314.9677	301.14166	356.56644	321.71567	290.9594
9	Ethanol	1654.7699	1691.5537	1805.0503	881.3973	1077.2028	969.6034	1427.1005	1175.3911	1471.594	1684.8501	1549.9733	1560.6982	1912.856	1490.327	1289.4972
10	(z)-2-penten-1-ol	470.43765	473.89417	462.2963	400.02832	409.7789	421.01813	260.76492	216.773	252.9029	198.9477	330.72037	298.33585	367.19305	478.82025	456.5386
**Aldehydes**																
1	Dodecanal	1407.4788	1459.9167	1116.7478	812.77496	968.8829	902.3966	1087.7976	802.1134	971.48553	849.5174	1196.4951	1183.7292	940.55786	908.31616	1131.0658
2	Decanal M	8699.794	8010.46	8047.6973	8610.486	8676.318	8617.019	9636.317	9388.772	9508.064	8893.1455	9098.017	8911.025	9608.741	9917.79	9654.65
3	Decanal D	7511.7256	8895.57	8719.257	5699.1753	5572.3125	5364.675	8126.1274	7006.5356	6936.6377	5147.067	7115.2905	8222.913	8874.975	8958.137	10107.844
4	Octanal	9440.306	8892.051	8484.624	9515.98	9380.806	9493.877	10170.29	10256.931	10251.3955	12208.275	11319.358	11149.552	11804.391	11645.4795	12846.371
5	Heptanal M	1621.6395	1635.345	1631.4409	1812.7441	1841.5199	1818.3654	1617.5197	1652.5449	1629.092	1162.9108	1236.1006	1331.9973	1503.9373	1588.5376	1420.416
6	Heptanal D	1228.394	1299.0985	1301.5426	1439.8347	1436.0894	1386.0636	1194.2733	1101.2142	1033.1473	458.27795	533.2198	659.90454	842.5949	902.23157	635.25824
7	Benzaldehyde	509.53207	497.36606	498.28967	363.62228	416.55228	355.69357	276.2605	248.24342	270.3251	314.47256	363.43503	353.83044	334.4689	277.05084	229.77696
8	(E)-2-Hexenal M	2550.5298	2477.7778	2472.6519	2574.0903	2586.0374	2628.7756	2416.494	1957.3685	1791.8845	1684.1455	2198.083	2130.8066	1944.9358	2144.8262	2004.1948
9	(E)-2-Hexenal D	5527.889	6040.881	5514.5073	6592.6562	6040.008	6084.022	3094.8396	1643.9751	1407.0851	953.2159	1974.67	1934.6329	1637.4652	2114.743	1648.279
10	Hexanal M	1300.0032	1270.8053	1297.829	1404.3142	1383.4451	1391.761	1319.3647	1136.3125	1144.2603	1029.5099	1181.1678	1122.5594	1479.637	1464.9412	1451.4136
11	Hexanal D	2134.8694	2228.3442	2278.6812	4106.28	3916.6445	4143.8154	1664.8696	1051.3123	1016.6646	598.9284	944.36035	848.7874	1966.5128	1999.0148	1680.2065
12	3-Methyl-2-butenal M	345.4288	341.31845	339.38864	525.99255	603.81006	588.0923	185.7025	147.26192	192.15846	121.71422	175.60275	139.29512	133.06134	186.1405	133.23909
13	3-Methyl-2-butenal D	227.26631	240.47977	206.77483	362.62247	499.84814	441.3287	106.370995	79.58225	101.45126	239.4609	128.37965	140.27907	191.4443	142.13269	104.41262
14	(E)-2-Pentenal M	1172.7883	1203.97	1138.2931	1061.8182	979.48724	988	493.2176	488.63113	539.0822	237.96277	376.8421	355.25238	380.5557	426.10608	404.82745
15	(E)-2-Pentenal D	1700.0886	1790.0975	1550.7128	1127.9045	1051.2996	984.5943	399.69186	353.4908	449.78427	163.07806	305.93762	305.4393	312.62845	381.28253	270.17273
16	2-Methylbutyraldehyde M	312.27612	322.0077	349.8026	376.40408	308.7498	395.937	253.5853	250.56998	285.19537	167.77562	197.65904	247.69115	195.23091	279.64084	302.02722
17	2-Methylbutyraldehyde D	389.04303	435.85986	462.82953	318.79877	271.52805	386.60855	198.80168	164.773	221.59433	246.16762	280.38675	361.00372	393.5057	472.39603	422.19568
18	Isovaleraldehyde M	347.82837	359.3088	363.65085	425.8966	370.08142	451.2348	257.4576	247.3166	297.9486	279.3774	300.6116	320.6143	292.50516	427.73752	460.6553
19	Isovaleraldehyde D	313.48544	352.55765	363.79053	328.80963	239.7307	394.41348	137.35262	110.01477	157.16805	125.15168	168.43265	217.91565	202.36293	395.21014	399.0888
20	Butanal M	570.20984	595.3925	609.9232	905.1358	970.0763	936.7332	548.07104	491.80835	721.7123	789.7538	770.1034	821.8082	862.1627	830.42883	759.2736
21	Butanal D	206.91766	223.62888	261.87268	353.72885	277.65707	380.29227	116.52786	98.48038	174.59976	123.62815	141.03766	170.06726	175.31392	254.68034	209.0506
22	2-Hexenal	122.56485	164.11914	120.143074	232.04639	165.9093	168.20094	26.436428	12.026369	10.312397	42.446194	98.670815	91.80223	70.98065	75.45285	52.8919
23	Pentanal	352.2339	388.61136	400.5425	215.23994	156.16823	185.06769	19.104437	21.373863	19.932858	25.455656	29.759628	26.544344	34.59049	43.204784	28.385277
**Monoterpenoids**																
1	α-Terpineol M	4460.97	4137.0425	4243.131	3791.988	3723.8767	3716.589	3943.6873	3682.4558	3684.6458	4199.142	4221.928	4157.499	3779.9617	3775.6926	4172.0674
2	α-Terpineol D	3410.2737	4758.0586	4392.0303	1570.214	1409.3864	1350.3241	1795.96	1538.2198	1420.178	2261.6333	3075.589	3818.5896	2049.1104	1842.3356	2895.0254
3	Linalool M	15599.248	14769.272	14714.8545	16043.767	15941.687	16024.772	15824.673	15848.707	15886.767	16181.513	16274.355	15457.382	15540.96	15565.031	15746.693
4	Linalool D	10057.215	10884.683	10953.289	7705.2744	7391.449	7581.9478	8647.736	7906.3774	7982.103	6894.1533	8944.031	10373.697	8422.943	8475.911	8877.815
5	Tetrahydrolinalool	451.72678	605.2574	654.715	171.54318	144.8941	161.86876	286.55704	291.3752	263.47556	91.275345	111.458954	123.015564	114.51871	122.047485	131.08075
6	α-Terpinene M	6924.325	6860.451	6886.2563	7050.988	7090.8125	7135.68	7019.419	7030.4966	7094.3135	6682.89	6910.1123	6937.8564	6719.36	6704.7563	6827.9146
7	α-Terpinene D	3576.0625	3910.1536	3960.4429	3332.9197	3378.3813	3272.061	3342.899	3317.8525	3280.2944	2369.3215	2903.0017	3610.434	4111.539	3901.5552	2957.6265
8	α-Pinene	14683.298	14984.147	14891.542	14499.992	14605.265	14657.141	14184.218	14108.8955	14152.544	7177.0537	7918.7114	8897.285	9502.641	9627.735	8504.138
9	β-Pinene	2871.252	2924.1787	2908.972	3048.5464	3022.7258	2954.6685	3053.5771	3071.7993	3073.2307	2902.8271	2928.4255	2879.6504	2790.6477	2834.554	3061.9185
10	Terpinolene	7051.394	7155.7876	7232.4497	7140.8257	6915.33	7005.882	6892.6104	6848.1553	6895.5244	4775.598	5321.067	5891.8857	6020.948	6003.04	5616.5557
11	(E)-Ocimene	3131.1123	3023.6526	2944.5337	3126.729	3137.7236	3166.839	3234.1155	3203.937	3260.2664	2965.9995	2944.34	2735.9402	2834.681	2987.6338	3036.8184
12	β-Myrcene	3638.0383	3342.937	3165.3916	3618.3086	3644.999	3576.2498	3765.1802	3791.9595	3789.2996	4546.491	4564.9673	4586.1123	4390.094	4477.748	4613.863
13	α-Thujene	9438.76	9460.445	9576.853	9262.678	9359.19	9208.234	9274.391	9165.41	9065.892	8573.966	9358.512	9952.316	10196.05	10351.964	9760.33
14	Norbornane,7,7-dimethyl-2-meth	5550.539	6083.2476	6435.472	4986.2803	5224.3286	5206.1987	4363.5723	4259.7656	4254.0654	1342.9921	1709.6932	2346.621	2695.2715	2629.458	1827.9001
15	γ-Terpinene	2792.9138	2517.9736	2501.5735	2611.3916	2725.7388	2737.0066	2886.1157	2854.2295	2839.4133	3377.594	3541.7131	3420.7734	3392.8071	3421.624	3535.3113
16	3-Carene	494.98553	543.6655	590.1681	470.88202	571.508	538.7204	499.7656	487.19647	489.36752	199.32222	238.78801	245.07892	234.71257	222.60367	206.30507
17	α-Phellandrene	2690.2278	3114.0867	3092.478	1959.5681	1925.0061	1942.1268	2130.978	1952.7979	1886.2482	1701.0916	2121.4558	2399.0178	1845.6271	2051.4148	2278.8557
18	Citronellal M	2296.2334	2173.7034	2166.165	2423.077	2417.3286	2461.4	2267.6228	2311.11	2272.6567	2304.9016	2315.6394	2284.5466	2349.4744	2481.0566	2493.4004
19	Citronellal D	4854.4697	6605.927	6784.6206	5009.854	5011.2534	4793.0303	4411.862	4348.9434	4171.731	2667.2195	4361.766	6160.573	5401.1855	5287.403	5859.9775
**Ketones**																
1	Cyclohexanone M	92.700485	78.96332	84.80669	77.15095	72.68827	89.954956	73.922966	72.247086	75.17353	363.49216	428.84528	481.8673	464.0293	492.31937	401.65976
2	Cyclohexanone D	32.349632	27.683819	29.220045	31.108587	27.30611	30.156382	29.20735	40.087894	42.833424	64.219986	86.84124	116.464386	119.46701	132.7979	95.420616
3	2-Hexanone	43.893547	54.037724	51.365196	43.788807	39.148384	44.122078	102.72722	140.19337	98.68351	63.84862	63.204296	63.826405	45.04572	70.714035	79.32198
4	3-Hydroxy-2-butanone M	1914.4525	1622.2997	1565.2277	2156.3606	2243.205	2141.357	2616.1018	2683.315	2567.0125	2586.8755	2575.03	2548.422	2456.55	2474.7437	2497.733
5	3-Hydroxy-2-butanone D	1919.9341	1381.3407	1345.8868	2413.0024	3177.837	2579.6133	4182.494	4119.8896	5391.3936	2637.2915	2693.478	3016.8315	3052.3267	2948.5203	2421.4517
6	2,3-Butanedione	589.51105	584.5405	621.3084	1270.4086	1453.2577	1387.9363	587.6638	483.6384	637.1341	472.6531	531.2265	510.41763	513.8932	769.294	785.7355
7	Acetone	3517.3208	3531.6643	3522.463	2806.2925	3039.4846	2855.5913	2289.1934	2033.234	2307.425	2950.409	3055.8435	3025.3188	2903.354	2815.7795	2725.818
8	1-Penten-3-one	239.46408	246.08191	199.50632	198.26527	131.63303	161.53549	119.74632	96.97589	74.12928	90.93255	139.73314	138.66032	114.94085	164.15407	172.67949
9	2-Butanone	81.99451	82.718185	83.467255	89.25984	98.98822	101.59092	86.53018	93.7003	85.15583	167.59152	156.39676	153.39096	150.75334	152.99103	160.02466
**Esters**																
1	Crotonic acid, hexyl ester	1865.5536	2187.0027	1624.5342	798.4601	871.2563	868.1299	1104.3691	881.8639	954.4347	979.6079	1376.9731	1988.1882	1054.7115	1058.0094	1303.155
2	Ethyl salicylate	1525.9872	1770.3075	1377.827	758.0675	748.2407	651.2299	850.4855	592.8914	684.47144	1106.2006	1246.67	1324.5193	877.6583	831.9365	752.4082
3	2-Nonynoic acid Methyl ester	1608.5974	1948.897	1551.0302	671.0993	741.9625	718.2335	1107.2893	729.3267	917.32404	919.08246	1351.5239	1633.7421	844.0073	848.86993	1130.7833
4	Ethyl acetate M	245.044	256.92755	271.97876	62.44253	61.706158	64.02002	142.5231	149.11554	241.75572	81.50571	78.18568	91.65623	97.54086	120.13038	63.08051
5	Ethyl acetate D	126.45303	130.76653	157.50449	18.196667	21.967405	16.815968	37.41537	42.51602	93.69395	35.149117	37.669292	43.842762	54.180553	74.58634	40.198986
6	Butyl acetate	21.453213	22.0658	17.83483	31.832266	23.75438	28.067875	21.862663	17.333334	17.676128	20.564487	21.069157	25.544527	57.12287	90.989685	40.325947
7	Styralyl acetate	746.8759	985.5782	973.16144	541.9166	494.9887	467.32394	458.17957	445.92786	440.76688	364.6697	383.67258	387.84644	389.48422	360.79742	408.27124
8	Methyl salicylate	730.5233	1020.8194	989.66956	677.3648	655.645	617.5313	359.8833	353.81458	341.18512	472.96417	599.906	725.42584	423.14154	385.34848	447.97827
**Others**																
1	Cis-Anethol	3709.292	4564.126	4075.882	2179.493	2319.5625	2134.063	2217.0542	1841.9135	1833.8419	1924.3206	2650.797	3161.5764	1860.9448	1622.1093	2034.2972
2	Methyl eugenol	1355.2693	1648.0442	1269.9706	733.2181	719.1413	690.8195	844.2739	579.8811	594.36096	803.9956	1090.8605	1249.7299	633.33795	606.85077	722.54065
3	4-Allylanisole	1216.939	1444.0847	1458.5963	1012.094	929.6456	829.5941	756.4392	750.3959	687.81055	875.4047	1008.40894	1142.8922	837.0943	751.88135	881.0672
4	2-Methoxy-3-isobutyl pyrazine	1968.23	2401.1316	2452.8237	1804.1648	1837.0825	1842.872	1689.8873	1622.6837	1576.0066	2044.0098	2738.6858	2776.2788	1741.7794	1717.2062	2272.8757
5	4-Ethylphenol	792.0803	1081.7606	1020.21313	405.148	340.10913	343.93066	370.46548	366.88202	344.82574	343.56247	454.82782	514.0773	478.55045	481.06744	550.6547
6	Allyl Propyl Disulfide	6675.406	6127.7666	5911.987	6830.2095	6761.3647	6794.0635	6925.3823	7048.163	7098.811	7850.9175	8026.952	8011.26	8167.101	8220.542	8150.6914
7	1-Mercaptopropane M	114.179085	104.22218	119.37179	140.59964	169.1214	149.47739	121.222244	117.85778	126.61173	534.2894	572.93634	597.6017	685.86804	743.813	659.3205
8	1-Mercaptopropane D	25.01764	27.464811	30.37539	17.349203	19.869377	19.717024	19.155222	17.352377	20.418482	197.41782	209.85362	223.31465	374.00452	382.0824	257.35287
9	2-Methylpropanoic acid	131.88377	155.89844	152.51175	66.85442	62.302876	65.137276	301.82727	411.0041	280.31058	20.002686	25.620705	44.99176	104.85381	83.80052	60.07471
**Unidentified**																
1		853.3389	1059.2949	848.1526	412.12134	492.45584	426.1505	587.0544	499.17523	444.95026	526.89716	774.63275	913.1153	632.1033	556.1267	912.34717
2		2445.2283	2999.1523	2928.835	1520.5056	1410.6654	1309.1094	1538.515	1370.0411	1272.4875	1935.6455	2483.688	3028.2993	2012.7742	1850.4325	2583.1428
3		1552.2109	1985.7411	1967.3223	1760.7156	1704.0784	1713.6735	1660.5054	1677.1499	1613.3077	1666.8883	2217.4763	2497.5488	2342.4092	2398.9353	2360.7073
4		6460.496	7172.7593	7337.834	5985.5894	5957.214	5910.4033	6325.902	6187.9014	6164.8203	5027.7554	5985.1006	7184.887	7403.6567	7224.1973	6363.8726
5		409.8075	441.65878	513.9916	374.58853	386.75455	383.77414	331.4282	338.94427	320.41434	106.58366	129.91272	171.25117	179.49094	185.26765	137.21931
6		201.79161	232.22096	231.20528	83.37838	77.43026	79.820305	478.1759	633.6046	453.98987	24.116219	32.755905	54.79314	121.40316	101.333824	73.11677
7		673.2005	632.5476	665.92883	919.603	1035.1406	978.26843	1360.5222	1522.3497	1787.5837	314.25354	282.2245	342.63248	305.80115	328.8985	329.39682
8		2249.1055	2225.6875	2422.0325	2670.419	3284.8237	2922.0107	2487.538	2556.6714	4230.7744	245.65977	240.09888	296.95514	303.4365	300.48782	264.71658
9		202.45181	212.29445	189.13995	151.68651	122.35537	123.88525	115.509	112.55716	105.209305	159.19942	164.94757	169.18806	143.51022	168.89923	169.23567
10		130.91254	130.11902	132.25832	182.72844	174.79655	182.32216	113.001526	104.24123	133.79454	98.55655	101.898796	110.75432	106.94549	103.32394	94.51285
11		134.60391	141.32649	148.42996	117.19758	118.37197	117.58164	58.07825	48.36257	55.862785	44.404564	53.993286	61.379234	56.872124	58.655922	50.48282
12		206.76213	227.56784	251.07465	127.77342	97.997925	123.65989	116.05176	85.159004	115.65818	53.542576	60.820606	73.17707	77.32552	93.62095	80.620155
